# Expression of Galpha14 in sweet-transducing taste cells of the posterior tongue

**DOI:** 10.1186/1471-2202-9-110

**Published:** 2008-11-13

**Authors:** Marco Tizzano, Gennady Dvoryanchikov, Jennell K Barrows, Soochong Kim, Nirupa Chaudhari, Thomas E Finger

**Affiliations:** 1 Rocky Mountain Taste & Smell Center, Univ. Colorado Denver Sch. Medicine, Aurora CO 80045 USA; 2 Dept. Cell & Devel. Biology, Univ. Colorado Denver Sch. Medicine, Aurora CO 80045 USA; 3 Department of Physiology and Biophysics, University of Miami Miller School of Medicine, Miami, FL 33136 USA; 4 Department of Physiology, Temple University Medical School, 3420 N Broad St, Philadelphia, PA 19140 USA; 5 Program in Neurosciences, University of Miami Miller School of Medicine, Miami, FL 33136 USA

## Abstract

**Background:**

"Type II"/Receptor cells express G protein-coupled receptors (GPCRs) for sweet, umami (T1Rs and mGluRs) or bitter (T2Rs), as well as the proteins for downstream signalling cascades. Transduction downstream of T1Rs and T2Rs relies on G-protein and PLCβ2-mediated release of stored Ca^2+^. Whereas Gαgus (gustducin) couples to the T2R (bitter) receptors, which Gα-subunit couples to the sweet (T1R2 + T1R3) receptor is presently not known. We utilized RT-PCR, immunocytochemistry and single-cell gene expression profiling to examine the expression of the Gαq family (q, 11, 14) in mouse taste buds.

**Results:**

By RT-PCR, Gα14 is expressed strongly and in a taste selective manner in posterior (vallate and foliate), but not anterior (fungiform and palate) taste fields. Gαq and Gα11, although detectable, are not expressed in a taste-selective fashion. Further, expression of Gα14 mRNA is limited to Type II/Receptor cells in taste buds. Immunocytochemistry on vallate papillae using a broad Gαq family antiserum reveals specific staining only in Type II taste cells (i.e. those expressing TrpM5 and PLCβ2). This staining persists in Gαq knockout mice and immunostaining with a Gα11-specific antiserum shows no immunoreactivity in taste buds. Taken together, these data show that Gα14 is the dominant Gαq family member detected. Immunoreactivity for Gα14 strongly correlates with expression of T1R3, the taste receptor subunit present in taste cells responsive to either umami or sweet. Single cell gene expression profiling confirms a tight correlation between the expression of Gα14 and both T1R2 and T1R3, the receptor combination that forms sweet taste receptors.

**Conclusion:**

Gα14 is co-expressed with the sweet taste receptor in posterior tongue, although not in anterior tongue. Thus, sweet taste transduction may rely on different downstream transduction elements in posterior and anterior taste fields.

## Background

Taste buds, the end-organs for gustation, detect and respond to a variety of macronutrient and aversive compounds to generate taste perception. Compounds that evoke bitter taste bind to one or more G protein coupled receptors (GPCRs) of the T2R family of taste receptors [1-3]. Amino acids and compounds that elicit umami taste bind to a variety of GPCRs including metabotropic glutamate receptors, mGluR4 and mGluR1, and the heterodimeric taste receptor, T1R1+T1R3 [4-7]. Sugars and a variety of other sweeteners bind to the heterodimeric receptor, T1R2+T1R3[5,8]. Most of these various taste GPCRs appear to all couple to a common transduction pathway that includes the heterotrimeric G protein subunits, Gβ3 and Gγ13, a phospholipase C, PLCβ2, and a transient receptor potential ion channel, TrpM5 [9-14].

In spite of the detailed exploration of the Gβγ-triggered signaling, much less is understood of which Gα subunits couple to various taste GPCRs, and which downstream signaling pathways they recruit. Taste buds are reported to express a number of different subunits including Gαgus (gustducin), Gαs, Gαi, Gαq, Gα14, Gα15 and two transducin isoforms, Gαt1 and Gαt2 [15-18]. Apart from Gαgus, it remains unknown which of these are expressed in the same cells as taste GPCRs and might be candidate signaling partners. In vitro, the sweet taste receptor, T1R2+T1R3, couples via Gαi to cAMP modulation[19]. Although Gαi subunits are expressed in taste buds[20], it is unclear which receptors activate them in situ. Biochemical and physiological studies have suggested that bitter taste transduction includes the involvement of Gαgus, although the exact mechanism of such involvement remained unclear[13,21,22]. While Gαgus-knockout mice are quite insensitive to bitter tastants, they are also somewhat compromised in their detection of sweet tastants[23]. Gustducin is co-expressed with the T1R2+T1R3 sweet receptor in the palate [24] and fungiform papillae[25] but not in the posterior gustatory fields. Furthermore, the direct functional role of Gαgus in sweet detection has not been demonstrated. The Gαgus, Gαi and Gαs subunits alter cAMP levels when activated, while members of the Gαq family trigger release of stored Ca^2+^. The primary cellular response triggered by tastants appears to be a Ca^2+ ^signal. Although much of this signal is produced via the action of Gβγ subunits[9], the contribution of the Gαq family has not been examined in taste buds.

Mammalian taste buds are composed of up to 100 cells. Though most mature cells in taste buds have a generally fusiform shape, they can be distinguished into several distinct types based on their functional properties and the expression of diagnostic mRNA and protein markers. Based on ultrastructural and other criteria, cells in rodent taste buds are classified as "Type I", the glial-like or supporting cells, "Type II", the primary receptor cells, and "Type III", the cells that show specialized chemical synapses[26]. Type II cells are characterized by the ubiquitous expression of PLCβ2 and TrpM5 [20,27-29]. Subsets of Type II cells express either T2Rs or T1Rs[8], an observation that suggested the segregated detection of tastants of the sweet and bitter qualities. Subsequent functional studies have demonstrated the equivalence of cell types identified by expression patterns and cells with particular response profiles. For instance, cells that express NCAM and SNAP25 were shown to be those that display voltage-gated calcium channels[29,30]. Cells that express TrpM5 or PLCβ2 are those that respond to bitter or sweet stimuli[29,31]. Here, we have used RT-PCR, immunocytochemistry and single-cell gene expression profiling to examine the expression of the Gαq family (αq, α11, α14)[32] in mouse taste buds and establish which of these are co-expressed with T1R2 and T1R3, subunits that constitute the sweet taste GPCR. The pattern of expression suggests that Gαgus is unlikely to be a signaling partner for T1R2+T1R3. Instead, the sweet receptor subunits consistently are co-expressed with Gα14.

## Results

### Gαq family members are differentially expressed in different taste fields

First, we used end-point RT-PCR to evaluate the expression of Gαq family subunits in taste buds to assess which members, if any, of the Gαq family (Gαq, Gα11 and Gα14) are expressed in taste buds. We analyzed taste buds obtained from four different oral taste fields (vallate, foliate, fungiform and palate) as well as non-taste lingual and palatal epithelium.

**Figure 1:**
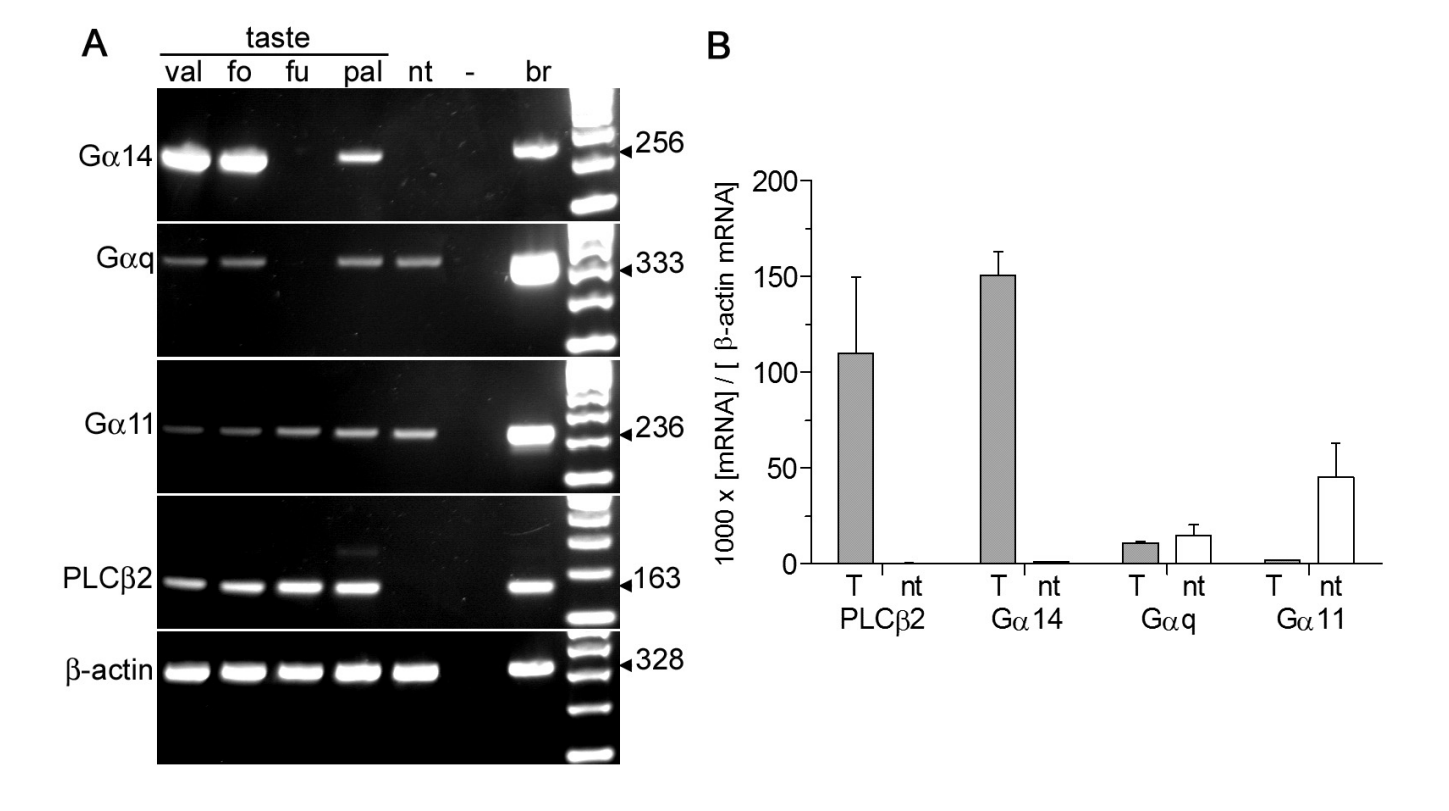
**Of Gαq family members only Gα14 shows a taste bud-selective pattern**. **A**. RT-PCR on isolated taste buds shows that Gα14, Gαq and Gα11 are all expressed in vallate (val), foliate (fo) and palate (pal). Fungiform (fu) taste buds express Gα11 but Gα14 and Gαq are not readily detected. Of these Gα subunits, only Gα14 is expressed in a taste-selective fashion (i.e. is absent from non-taste lingual epithelium [nt]). Brain (br) cDNA and water in place of cDNA (-) served as positive and negative controls run in parallel for all PCRs. Predicted sizes of products (in basepairs) are to the right. All templates were also analyzed for PLCβ2, a gene expressed in all taste buds and for β-actin, expressed in all cells. **B**. Quantitative RT-PCR shows that only Gα14 is prominently expressed in a taste-selective manner. mRNA from three samples of CV taste buds (T = gray bars) and of nontaste lingual epithelium (nt = white bars) were analyzed for expression of Gαq, Gα11 and Gα14. The same samples were also analyzed in parallel for β-actin as a normalization control and PLCβ2 as a taste-selective marker.

As shown in Fig. 1A, Gα14 is strongly expressed in vallate and foliate taste buds, with somewhat lower expression in the palate. Under parallel conditions, expression of Gα14 was negligible in the fungiform field. Gαq also was not detected in fungiform papillae, and its mRNA was seen in taste buds from vallate, foliate and palate. Finally, we observed that Gα11 appeared to be expressed similarly in taste buds of all taste fields. Of these three Gα subunits, Gαq and Gα11 were detected in non-taste mRNA at roughly similar levels as in taste buds. In contrast, Gα14 was expressed in a highly taste-selective manner. We also tested expression of the distantly related subunit, Gα15. We detected RT-PCR product for Gα15 prominently in the nontaste epithelium samples and very little in vallate taste bud samples (data not shown). Thus, we did not investigate this subunit further. The taste selective expression pattern and apparently high mRNA level of Gα14 suggested that it may be the principal Gαq family member playing a taste-specific role in murine taste buds.

To test this, we undertook quantitative RT-PCR experiments to compare expression levels of the Gαq family members in taste buds (Fig. 1B). The concentration of Gα14 mRNA in vallate taste buds was comparable to that of PLCβ2 mRNA. Neither mRNA was expressed in nontaste epithelium. In contrast, Gαq mRNA was found at similar concentration in CV taste buds and in nontaste epithelium while Gα11 was at much higher concentration in nontaste epithelium than in taste epithelium. Thus, neither Gα11 or Gαq are expressed in a taste-selective manner. Further, the mRNAs for Gαq and Gα11 are expressed at 14- and 80-fold lower concentrations respectively than Gα14 mRNA. These data support our interpretation from end-point RT-PCR, that the only Gα subunit of this family that is likely to have a taste-selective role is Gα14.

**Figure 2:**
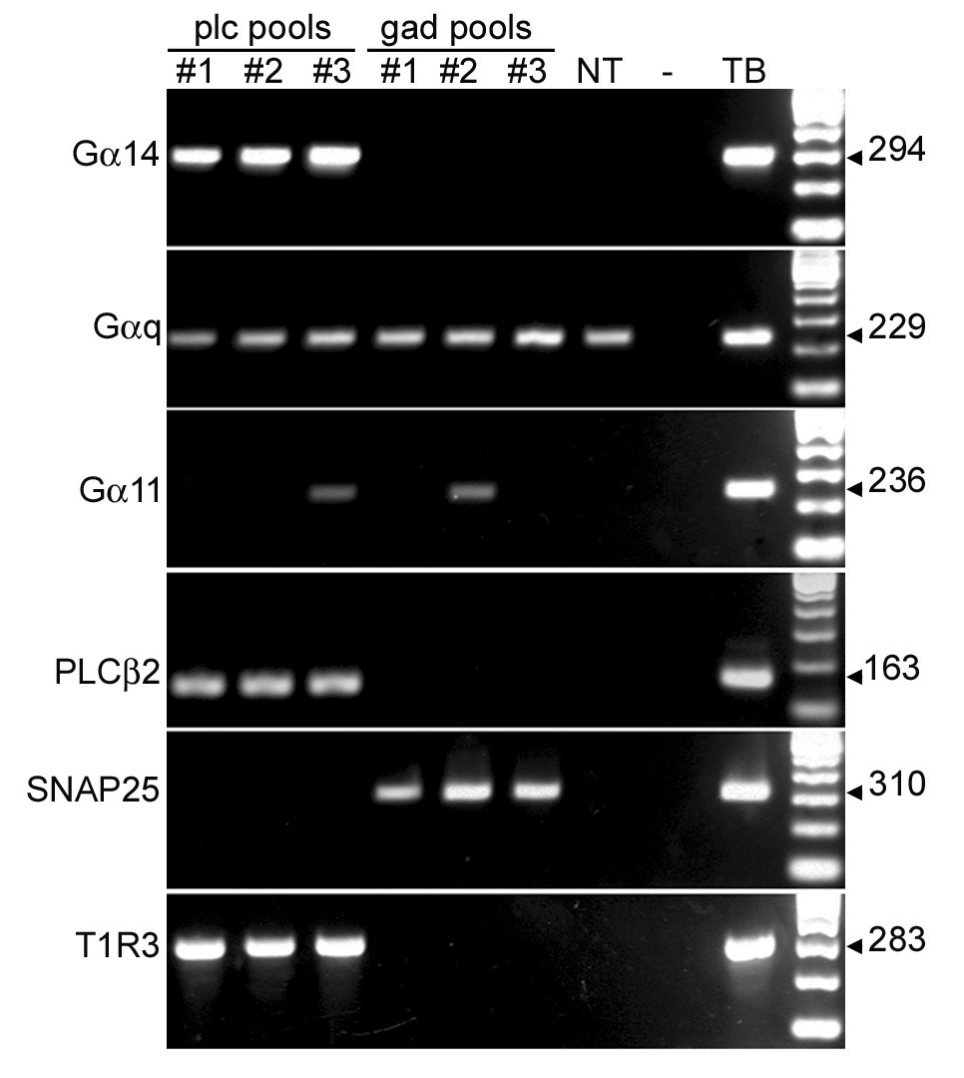
**Gα14 is expressed only in Type II/Receptor cells**. Three pools (#1, 2, 3), each containing ten individual GFP(+) taste cells were collected from PLCβ2-GFP mice (plc pools) or from GAD-GFP mice (gad pools). Amplified mRNAs from these pools represent Type II and Type III cells respectively and were analyzed by RT-PCR. RNA from a cluster of non-taste epithelial cells (NT) and a taste bud (TB) were amplified and analyzed in parallel. A negative control (-) reaction was run with no cDNA. RT-PCR for PLCβ2 (Type II cells), T1R3 (Type II cells) and SNAP25 (Type III cells) [29] confirmed that the pools were not cross-contaminated with cells of the opposing type.

Taste buds contain two cell types that have been functionally defined to date, Type II/Receptor cells and Type III/Presynaptic cells. To assess whether Gαq, Gα11 or Gα14 are selectively expressed in these two cell types, we used PLCβ2-GFP and GAD-GFP transgenic mice that respectively illuminate Type II and III taste cells [33,34]. Individual GFP-labeled cells from each strain were harvested to produce 3 pools, each of 10 cells, representing Type II and Type III cells respectively. We examined expression of the Gα subunits by RT-PCR on amplified RNA from these 6 pools (Fig. 2). Gα14 expression was limited to Type II cells and was detected in each of the three pools. In contrast, Gαq expression was less prominent, and was found in Type II, Type III and non-taste epithelial cells, consistent with the qRT-PCR data of Fig. 1B. Gα11 was only sporadically detected in the pools of identified taste cells, consistent with the low level seen in qRT-PCR with whole vallate taste buds. The absence of Gα11 in the isolated non-taste cells may reflect the heterogeneity of the non-taste epithelium, with some regions expressing Gα11 and others not. In summary, RT-PCR analyses suggested that Gα14, expressed only in taste buds, and only in Type II/Receptor cells within taste buds, was a reasonable candidate for coupling to a taste GPCR.

### Immunoreactivity to Gαq-family in Gαgus-negative Type II cells

**Figure 3:**
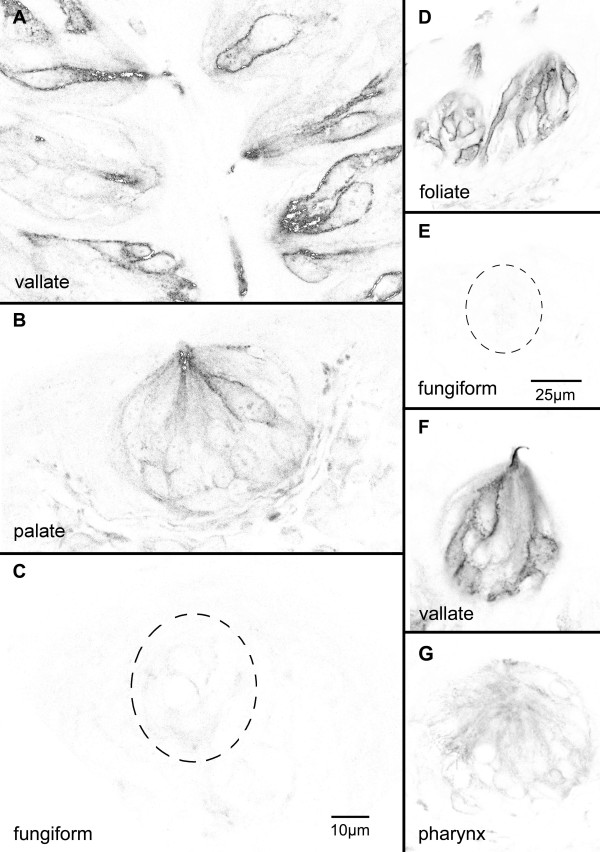
**Gαq-family immunoreactivity in different taste fields**. Inverted grayscale images of single confocal image plane of immunofluorescence showing Gαq/11/14-immunoreactivity in different taste fields. Acquisition and display parameters are matched to permit comparison of fluorescence intensity between image sets (A-C), (D-E) and (F-G). The heavy staining is largely membrane-associated and outlines the entire cell. Vallate (A & F) and foliate field (D) taste buds each contain several Gαq/11/14-immunoreactive taste cells. Rare immunoreactive taste cells occur in palatal taste buds (B) but essentially no Gq/11/14immunoreactivity is detected in fungiform field taste buds (C & E). Similarly, pharyngeal taste buds exhibit little or no specific immunofluorescence. Scale bar equals 10 μm for panels A-C and 25 μm in D-G.

Next, we used an antiserum that recognizes Gαq, Gα11 and Gα14 to perform fluorescent immunocytochemistry on cryosections of taste epithelia. The Gq/11/14 antibody reacted with a subset of taste cells in posterior (vallate and foliate) but not anterior (fungiform and palate) oral taste fields (Fig. 3). Immunoreactive cells in each case were elongate and spindle-shaped, as is typical for mature taste cells. Immunoreactivity appeared to be membrane-associated, largely encircling the taste cell profiles of immunoreactive cells. Relative to the signal in the vallate papilla, immunoreactivity to Gq/11/14 antibody occurred only rarely in cells of taste buds in the soft palate, and was essentially absent in taste buds of the fungiform papillae and pharynx (Fig. 3).

### Gαq-family-immunoreactivity is not due to Gαq nor Gα11

**Figure 4:**
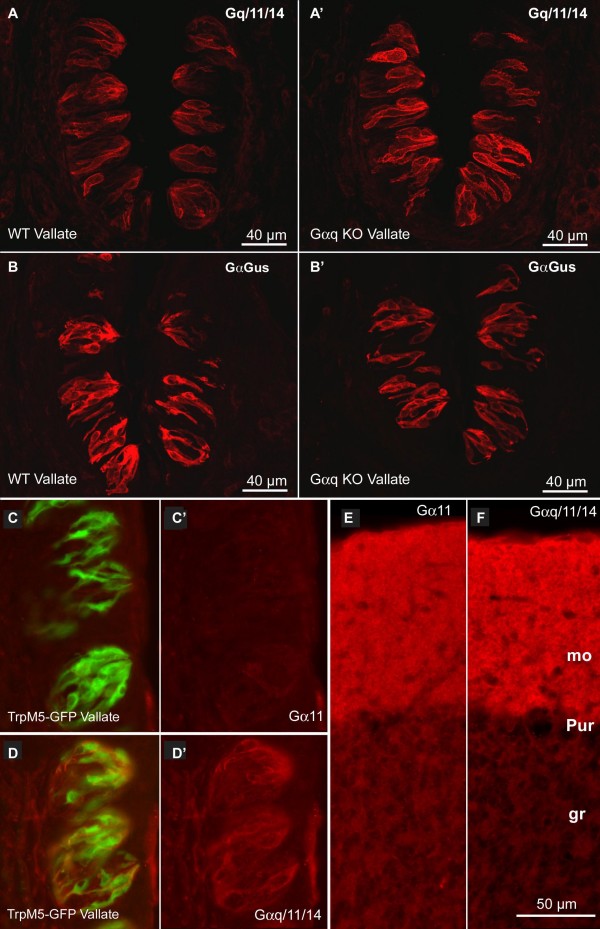
**Gα14 subunit expression in vallate papilla**. Using Gαq-null mice and Gα11-specific anitserum, we show that the immunoreactivity in taste buds revealed by Gq/11/14 antiserum must be due to Gα14. **(A) **Gq/11/14 immunoreactivity in vallate papilla of (A) WT and (A') Gαq null mice. The continued immunoreactivity for Gq/11/14 in Gαq-null mice demonstrates that members of the Gαq-family other than Gαq are responsible for most of the Gq/11/14 antibody immunoreactivity. **(B) **Gαgus immunoreactivity in (B) WT and (B') Gαq null mice. Continued immunoreactivity for Gαgus shows that other Gα expression is not altered in the Gαq null animals. Scale bars = 40 μM for A-D. **C-F**: Micrographs of immunostaining of TrpM5-GFP mice with an antiserum specific for Gα11 (C, C', E) or the broad Gq class antiserum (Gq/11/14) which reacts with Gαq, Gα11 and Gα14 (D, D', F). **C**, **C'**: Section through taste buds of the vallate papilla stained with Gα11-specific antiserum (red). **C**. shows the combined image of TrpM5-GFP (green) and Gα11 (red); **C' **shows only the red channel of this same image. No specific Gα11 staining is evident. This exposure is matched to that of panel E. **E**. Gα11 staining of the cerebellum showing evident immunoreactivity of the molecular layer corresponding to the demonstrated presence of Gα11 in Purkinje cell dendrites[35,56]. **D**, **D'**: Sections through taste buds of the vallate papilla stained with Gq/11/14 antiserum (red). **D **shows the combined image of TrpM5-GFP (green) and Gq/11/14 (red); **D' **shows only the red channel of this same image. Many taste cells in each taste bud show clear membrane-associated immunoreactivity similar to that shown in Fig. 3. **F**. Gq/11/14 staining of the cerebellum showing evident immunoreactivity of the molecular layer corresponding to the demonstrated presence of Gα11 in Purkinje cell dendrites [35,56]. Since Purkinje cells express both Gαq and Gα11, and higher levels of Gαq than Gα11, immunoreactivity with the broad Gq/11/14 antiserum is much greater than that obtained with the specific Gα11 antiserum. Exposure for this panel is 20% of that for panels **D**, **D'**. Scale bar in **F **(50 μm) also applies to panels C, D & E.

To assess whether the Gαq-family immunoreactivity was attributable to Gαq, we examined immunoreactivity with the same Gq/11/14 antibody in Gαq-KO mice. In vallate taste buds, staining with anti-Gq/11/14 was essentially no different in Gαq-KO mice compared to WT controls (Fig. 4A, A'). Staining with anti-Gαgus antibody also shows no difference in the Gαq-KO compared to WT animals (Fig. 4B, B') indicating that the Gαq-KO did not disrupt expression of other taste-related G-proteins. The robust staining by the Gq/11/14 antibody in Gαq-KO mice indicates that the bulk of the staining observed with Gq/11/14 antibody must be attributable to Gα11 or Gα14. Because the RT-PCR data (see Figs. 1 &2) showed that Gα11 is expressed at very low levels, the combined analyses suggest that Gα14 is the principal Gαq family subunit in Type II (Receptor) cells.

To further test whether Gα11 is present in taste buds, we utilized an antiserum directed against the N-terminal region of Gα11 which shares no sequence similarity to the N-terminal region of either Gαq or Gα14. This Gα11-specific antiserum does not stain taste buds (Fig. 4C–C') although it does stain the cerebellar molecular layer (Fig. 4E) in which Gα11 is detectable by immunocytochemistry[35]. These results essentially rule out Gα11 as the source of the taste bud immunoreactivity for the Gq/11/14 antibody. Since this Gq/11/14 antibody exhibits staining in Gαq knockout mice, we conclude that the staining is attributable to neither Gαq nor Gα11 leaving only Gα14 as the possible source of the immunoreactivity. These results are entirely consistent with our RT-PCR data (above) showing that Gα14 mRNA is the predominant Gαq family member isoform expressed in a taste-specific manner.

### Gαq-family-immunoreactivity is in Type II Taste (Receptor) Cells

Taste buds (TBs) comprise at least three different types of mature cells, so we utilized type-specific markers to test whether Gαq-family expression correlates with a specific cell type. Type II (receptor) cells express the GPCR taste receptors (T1Rs and T2Rs in different cells), TrpM5 and PLCβ2 [20,27-29]. Thus, we performed the first set of immunocytochemistry using tissues from TrpM5-GFP mice in which all GFP labeled taste cells express the TrpM5 protein[28]. In vallate taste buds, PLCβ2 antibody stained over 92% (25 of 27 cells) of TrpM5-GFP-labeled cells, confirming the identification of Type II cells with these markers. Further, in vallate taste buds, immunoreactivity to PGP9.5 antibody, which stains mostly Type III cells [26]) was detectable only in a small percentage of TrpM5-GFP positive cells (~4%; 1 of 27 cells).

**Figure 5:**
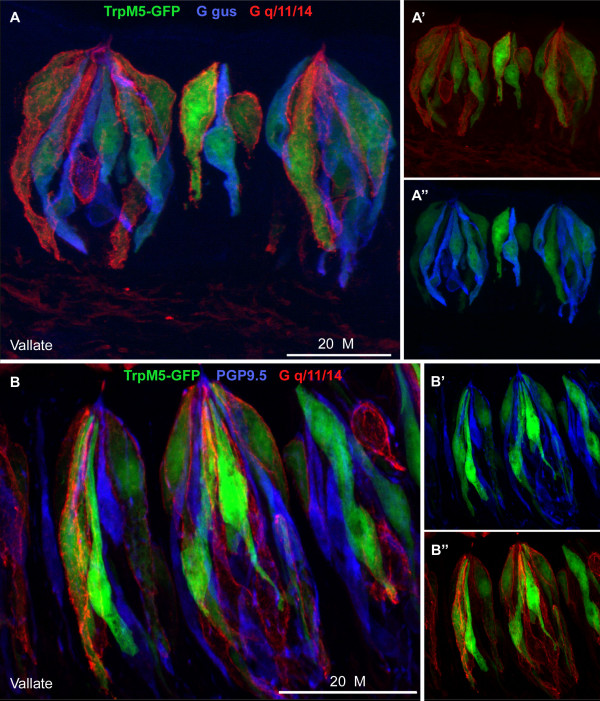
**Gq/11/14 immunoreactivity in Type II cells**. (A) Co-localization of Gq/11/14 (red), Gαgus (blue) in the vallate papilla of a TrpM5-GFP (green) animal. TrpM5 is a marker of Type II taste cells. Gq/11/14 stains about half (14 of 27) of the TrpM5-GFP cells. Usually the Gαq-family immunoreactive cells are different from those positive for Gαgus (blue). A', A", represent single staining of TrpM5-GFP TBs used to obtain the merged pictures A. The figure in Additional file 1 shows single plane confocal images from this image set for comparison. (B) PGP9.5 (blue), a marker of Type III cells, does not co-localize with either Gq/11/14 (red) or TrpM5 (green). The figure in Additional file 2 shows single plane confocal images from this image set for comparison. Scale bar 20 μM.

Next, we compared immunostaining for Gq/11/14 with staining for markers of Type II and Type III taste cells. In vallate and foliate taste buds of TrpM5-GFP mice, the Gq/11/14 antibody stained a subset of TrpM5-GFP cells (Fig. 5A). Nearly all Gq/11/14 positive cells expressed TrpM5-GFP (13/14), i.e. Gαq-family protein (presumed Gα14) is expressed only in Type II taste cells. We also immunostained the sections for Gαgus and viewed them for fluorescence in three colors: GFP (green) for TrpM5, Alexa647 (pseudo-colored blue) for Gαgus and Rhodamine RedX (red) for Gq/11/14. Most taste cells that were immunoreactive for Gq/11/14 lacked expression of Gαgus (only 4 of 27 cells co-express these G protein alpha subunits; see Table 1). In contrast, antiserum to PGP9.5 (Type III cell marker) stained none of the Gq/11/14-positive cells (Fig. 5B). We conclude that Gαq-family immunoreactivity is not in Type III cells, but is common in the TrpM5-immunoreactive (Type II) receptor cell population.

**Table 1:** T1R & G-protein co-localization in Taste Buds

	**Immunocytochem**.			**Single Cell RT-PCR**	
**TrpM5 with**		%	**TrpM5 with**		%
**Gq/11/14**	14/27	51.9	**Gα14**	13/21	61.9
**Gαgus**	17/27	63.0	**Gαgus**	15/21	71.4
**Gq/11/14 + Gαgus**	4/27	14.8	**Gα14 + Gαgus**	7/21	33.3

**Gq/11/14 with**			**Gα14 with**		
**T1R3(GFP)**	30/35	85.7	**T1R3**	13/14	92.9
			**T1R2**	10/14	71.4
			**T1R3 + T1R2**	10/14	71.4

**T1R3(GFP)**			**T1R3+T1R2 with**		
(includes T1R1 & TT1R2)			**Gαq**	5/10	50.0
**Gq/11/14**	30/41	73.1	**Gα14**	10/10	100.0
**Gαgus**	11/41	26.8	**Gαgus**	4/10	40.0

Results from immunocytochemistry are not significantly different (p > 0.05) from data from single cell RT-PCR (Fisher's Exact Test). The immunocytochemical data and RT-PCR data showing co-expression of gustducin with T1Rs is not directly comparable since some cells express only T1R3 and not T1R2+T1R3

### Gαq-family immunoreactivity in T1R3-expressing cells

**Figure 6:**
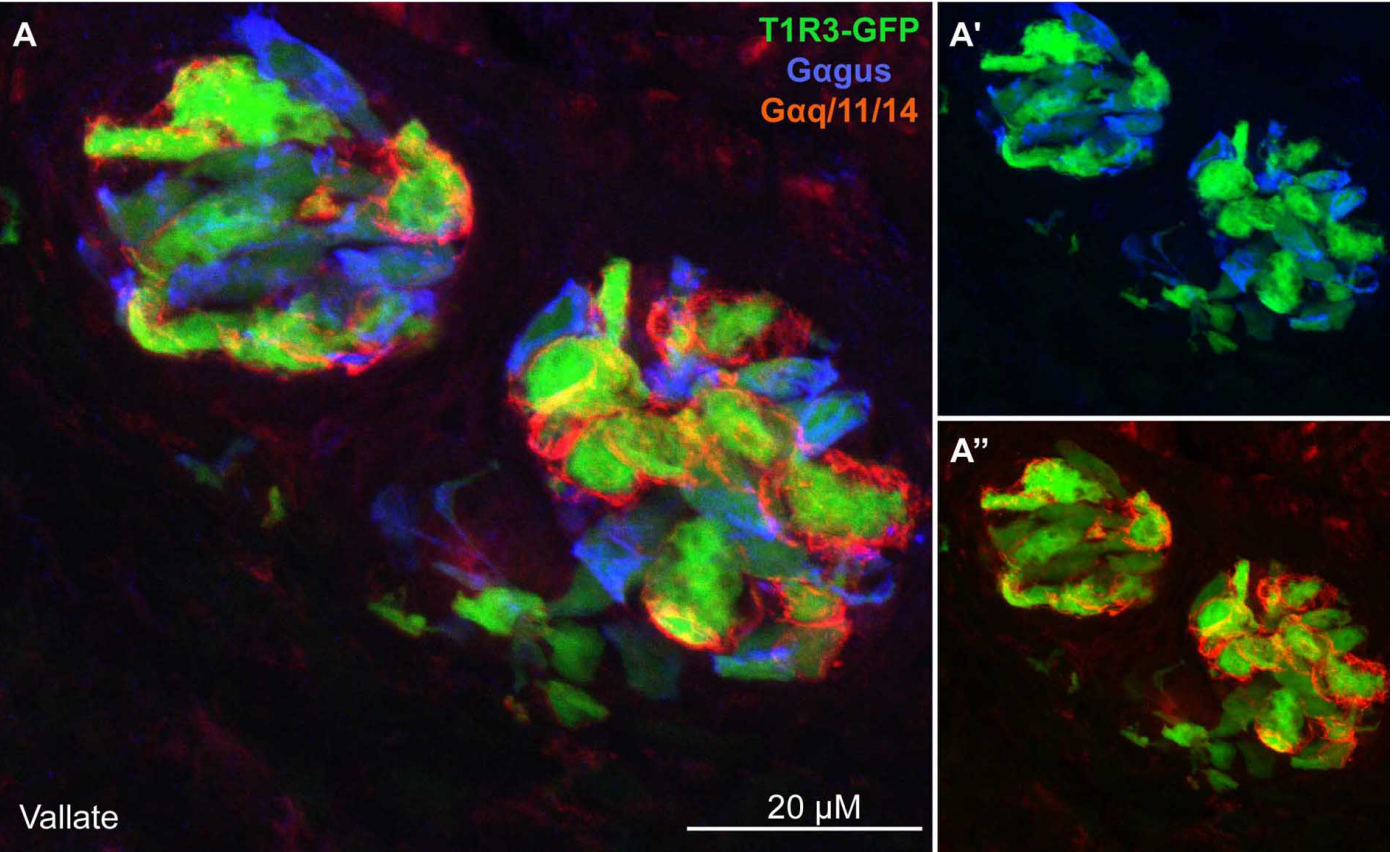
**Gαq-family immunoreactivity co-localizes with T1R3-GFP**. In vallate papilla TBs, the Gq/11/14 antibody (red) stains most of T1R3-GFP (green) cells (30 of 41). Fewer cells are strongly positive for Gαgus (blue). The large majority of Gq/11/14-IR cells (30 of 35) exhibit T1R3-GFP. Only about half of GαGus IR cells show T1R3-GFP expression). The figure in Additional file 3 shows single plane confocal images from this image set for comparison. Scale bar 20 μM.

Type II cells in taste buds express G protein coupled taste receptors for sweet, bitter or umami qualities. The taste receptors for sweet (T1R2 and T1R3) and for bitter (T2Rs) are expressed in separate subsets of Type II cells[1,8]. In taste membranes, bitter (T2R) taste receptors couple functionally to Gαgus [9,21,36,37]. Yet to date, it is unclear which Gα subunits natively couple to the other class of taste receptors, the T1Rs. Because T1R3 appears to be an obligatory subunit in these dimeric receptors, we used T1R3-GFP transgenic mice, to ask whether the Gαq family subunits are co-expressed with T1R3. By double-immunostaining (Fig. 6), in vallate papillae we found that the Gq/11/14 antibody stained the majority of T1R3-GFP cells (85.7%; 30 of 35 T1R3-GFP cells; see Table 1). In contrast, a smaller fraction of T1R3-GFP cells were strongly positive for Gαgus (~26.8%; 11 of 41 T1R3-GFP cells; see Table 1) (Fig. 6). Thus Gαgus expression does not correlate with T1R3 expression and therefore cannot be an obligatory partner in taste buds in vallate papillae.

### Single-cell RT-PCR shows co-expression of Gα14 with T1R2/T1R3

**Figure 7:**
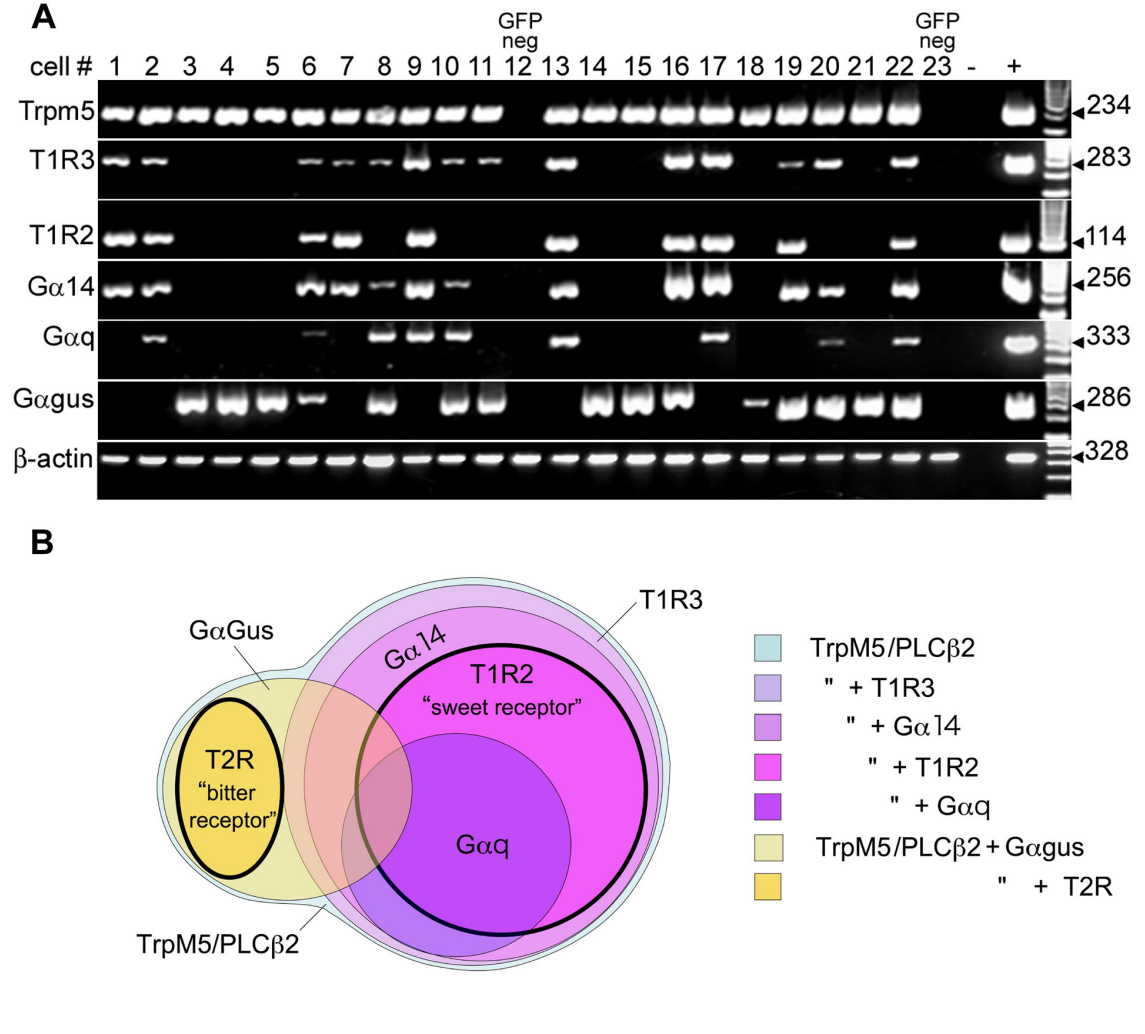
**Single-cell expression profiling on Type II cells**. **A**: mRNA was purified from 21 individual PLCβ2-GFP cells and two GFP negative cells (# 12 and # 23) and reverse transcribed. Each cDNA was divided into 7 individual tubes and was used to assay the expression of three Gα subunits (Gαgus, Gαq, Gα14), sweet receptor subunits (T1R2, T1R3) and TrpM5. β-actin served as a positive control for all samples. All PLCβ2-GFP positive cells displayed PCR product for TrpM5. In contrast, the Gα subunits and sweet receptors were expressed in only a fraction of PLCβ2-GFP cells. **B**. Venn diagram showing co-localization patterns based on the single cell RT-PCR analysis and previous studies on T2R localization patterns [1]. T2Rs do not co-localize with T1Rs and in the vallate papilla, always co-localize with Gα Gustducin. In our single cell RT-PCR study, all T1R2+T1R3 (sweet receptor) expressing taste cells express Gα14. A subset of these also express Gαq. The sweet receptive cells are themselves a subset of the Type II (receptor) taste cells identified by expression of PLCβ2 and TrpM5. Another set of TrpM5/PLCβ2-expressing cells express the T2R family of bitter receptors. These T2R-expressing cells invariably express GαGustducin [1].

We used transgenic PLCβ2-GFP mice to isolate individual GFP-labeled (Type II/Receptor) cells and performed single cell-RT-PCR as an independent test of the expression pattern of Gαq-family subunits and sweet receptors (T1R2, T1R3). Because Gα11 expression was limited to a low concentration of mRNA (Fig. 1B) and only in a few cells (Fig. 2), we did not include it in this analysis. For this detailed analysis, we collected 21 individual PLCβ2-GFP-expressing (i.e. Type II/Receptor) cells and 2 PLCβ2-GFP-negative cells. As expected, TrpM5 was expressed in all 21 PLCβ2-GFP cells, and was not detected in GFP-negative cells (Fig. 7). The taste receptors, T1R2 and T1R3 were detected in approximately half of GFP-positive cells (10 of 21 cells). All 10 of these T1R2+T1R3-expressing cells also expressed Gα14 (Table 1). In contrast, Gαq was detected in 50%, and Gαgus in only 40% of these T1R2+T1R3-expressing cells. In summary, Gα14 is always found in presumptive sweet-sensing Type II/Receptor cells. Additionally, Gα14 was not detected in the absence of T1R3. Thus, we propose that Gα14 may be the principal Gα subunit coupled to the sweet receptors.

## Discussion

Using the complementary techniques of immunocytochemistry and gene expression profiling via single-cell RT-PCR, we have explored the pattern of expression of the Gαq family of heterotrimeric G protein subunits. We show that while Gαq and Gα11 are expressed at low levels in taste buds, Gα14 is expressed in a highly taste bud-selective manner. This observation is consistent with many previous reports of the ubiquitous distribution of Gαq and Gα11, while Gα14 is restricted to a few highly specialized cell types[38]. Further, we show that Gα14 is limited to Type II taste cells, i.e. those that express taste GPCRs.

Mammalian taste buds use specialized taste GPCRs (including the T1R and T2R families) to detect bitter, umami and sweet tastants. Both families of taste receptors activate the downstream signalling elements, PLCβ2 and IP3R3[9,12,20,27,39]. The T2R receptors couple to Gαgus[22,40] but the Gα subunits activated in situ by the T1R receptors are not yet defined. Whereas Gαgus is substantially co-expressed with T1R receptors in the anterior taste fields, such is not the case for posterior taste fields[24,25] where only a fraction of T1R3-expressing taste cells co-express Gαgus. Hence, Gαgus is unlikely to be the Gα subunit associated with the T1R receptors in the posterior tongue. We report here the obligatory co-expression of Gα14 with the sweet receptor T1R2+T1R3 in foliate and vallate taste fields. Accordingly, we suggest that sweet transduction in posterior, but not anterior gustatory fields involves Gα14. Whether Gα14 couples directly to the sweet taste receptor or to other GPCRs intimately associated with sweet detection remains to be determined.

The different taste receptors are not homogeneous across the taste fields of the tongue but show regional differences[25,41]. The T1R1+T1R3 umami receptor is much more prevalent in the anterior taste fields (fungiform and palate) than in the posterior lingual fields (foliate and vallate). In posterior lingual taste fields, T1R3 more commonly partners with T1R2 forming a sweet receptor. Our single cell RT-PCR data show that over 70% (10/14) of the T1R3-expressing cells also express T1R2. With in situ hybridization, two groups reported previously that 92%[25] and 98%[42] of the T1R3 expressing cells of the vallate papilla co-express T1R2. The lower percentage of co-localization we report may reflect differences in technique, given the higher sensitivity of single cell RT-PCR over in situ hybridization. Most of the T1R3-positive, non T1R2-expressing cells in our profiling displayed low levels of T1R3 (e.g. cells 8, 10, 11 of Fig. 6) and may not have been detected with in situ hybridization.

The T1R2+T1R3 heterodimer can functionally couple to a variety of Gαi/o subunits in heterologous systems[19,43]. Since sweeteners activate adenylyl cyclase in vallate taste buds[44], Margolskee[45] has postulated that the T1R2+T1R3 receptor may couple to Gαs. Indeed, Kusakabe et al[18] showed the expression of Gαs, Gαi2 and Gαi3 in vallate taste buds, but not the tight association with sweet receptors. In contrast, our data show clearly that Gα14, a member of the Gαq family is consistently co-expressed with T1R2+T1R3 in posterior taste buds. Because sweet taste receptors are co-expressed with PLCβ2[46], and mice in which PLCβ2 is knocked out show a severe deficit of sweet signaling[12], it is widely accepted that sweet transduction occurs via Gβγ-mediated activation of PLCβ2. Yet PLCβ2 also can be activated robustly by members of the Gαq family[47]. Our study is the first demonstration of a consistent association of Gα14 (a Gαq family member) with taste receptors that activate PLCβ2.

The expression of Gαgus in association with the sweet receptor in the palate and fungiform papilla is likely to account for the profound decrease of intake of sucrose and sweeteners in long-term taste behaviours in Gαgus knockout mice[36,48,49]. On the basis of these previous results along with our current findings, Gαgus knockout would be predicted to affect only the palatal and fungiform taste fields where Gαgus is co-expressed with the sweet receptors. Transection of the gustatory nerves innervating only anterior taste fields results in near-total elimination of sweet taste preference in some behavioural assays despite the presence of an intact posterior lingual taste system[50]. Thus the loss of sweet-driven taste behaviours reported in Gαgus knockouts is likely attributable to loss of function in the sweet-detecting taste buds of the anterior taste fields. Indeed more rigorous behavioural analysis of gustducin null mice reveals substantial residual sweet-guided taste behavior[49]. Electrophysiological analysis of taste nerves of Gαgus-null mice reveals a profound loss of sweet-evoked activity in anterior tongue but only partial loss in posterior. This is consistent with our finding that relatively few sweet receptive taste cells also express Gαgus. The degree of functional loss as measured by the magnitude of the glossopharyngeal nerve response in Gαgus null mice, is, however, greater than would be predicted on the basis of the co-expression patterns[51]. Perhaps Gαgus-expressing sweet-receptive cells disproportionately activate gustatory nerve fibers, or act synergistically with other cells within a taste bud to effect activation of the nerve fibers. Indeed, Roper[52] suggests the likelihood of intrabud intercellular communication playing an essential role in transmission of taste information.

## Conclusion

The sweet-receptive taste cells of the posterior tongue express Gα14 while those in anterior taste buds express Gαgus. These findings show that even within a single sensory system, the same receptor may couple with different G-protein alpha subunits in different functional parts of the system. Our results also account for the residual behavioural and neural activity to sweet tastants in Gαgus-null animals.

## Methods

### Animals

All experiments were performed under protocols approved by the Animal Care and Use Committees of the CU Denver School of Medicine and the University of Miami School of Medicine. We used three strains of adult transgenic mice in which GFP is expressed from the promoters of T1R3, TRPM5, PLCβ2, or GAD1[31,34,53]. In taste buds from each of these strains, previous studies have shown that all cells expressing the relevant endogenous proteins also express GFP [31,33,34]. Gαq-deficient mice and littermate controls were obtained from Satya Kunapuli (Temple University, Philadelphia, PA) [54] with permission from Stefan Offermanns (University of Heidelberg, Heidelberg, Germany). This Gαq-null line was generated by replacement of sequence coding for amino acids 246–297 of Gαq by the neo gene and is described in detail in Cho et al[55]. The genotype of Gq-null mice was determined by PCR and was confirmed by platelet aggregation assay.

### Immunofluorescence

We used a Gq-family antibody that is labelled and sold as anti-Gαq/11 (Santa Cruz Biotechnology; sc# 392) although the manufacturer indicates that it likely will react with Gα14. The antigenic peptide, VFAAVKDTILQLNLKEYNLV, is located near the C-terminus, is 100% identical between Gαq and Gα11 and is 90% identical/100% similar in Gα14 (all sequences from mouse). In contrast, Gα15 is only 45% identical/75% similar in this region. Thus, the antibody likely reacts with Gαq, Gα11 and Gα14, but not Gα15. To test the specificity of this broad Gαq family antibody, we also used an affinity-purified Gα11 specific antiserum (Santa Cruz Biotechnology; sc# 394) directed against an N-terminal peptide (aa 13–29 from mouse sequence) that lacks any sequence similarity to either Gαq or Gα14. The cerebellum was used as a positive control tissue for these antisera since Purkinje cells express both Gαq and Gα11 but not Gα14 [35,56].

Mice were perfusion-fixed in 4% paraformaldehyde (PFA) in 0.1 M phosphate buffer (PB). Taste tissues (vallate, foliate and fungiform papillae and soft palate) were post-fixed (4°C, 60 min); 14 μm cryosections were washed with phosphate-buffered saline (PBS), blocked with 1% normal goat serum, and incubated with rabbit anti-Gαq-family antibody (1:500); at 4°C overnight. The secondary antiserum used was Rhodamine RedTM-X-conjugated AffiniPure™ Fab Fragment goat anti-Rabbit IgG (H+L) (1:100; Jackson ImmunoResearch Lab.; 111-297-003).

To examine co-expression of the Gαq family with other proteins in taste buds, and to avoid cross reactivity of multiple rabbit primary antibodies with a common secondary antibody, we used the Zenon Rabbit IgG Labeling Kit (Invitrogen, Z25308). For this, each primary antibody other than Gαq/11/14 was pre-conjugated to Alexa Fluor 647 so that it did not require a secondary antibody for visualization. After the binding of the first primary and secondary antibodies was complete, the primary antibody-Zenon647 complex was applied to the slides for 80 min at RT in the dark, washed in PB with 0.2% Triton X-100 and postfixed in PFA/PB. Slides were then coverslipped with Fluormount-G. Omission of primary antibodies (detected with Rhodamine Red-X or Zenon) resulted in no apparent fluorescent signal. The primary antibodies used as Zenon complexes were rabbit anti-PLCβ2 (1:200; Santa Cruz Biotechnology; sc# 206); rabbit anti-Gαgus (1:200: Santa Cruz Biotechnology; sc# 395) and rabbit anti-PGP9.5 (Ubiquitin C-terminal hydrolase-L1; 1:200; AbD Serotec; 7863-0504).

All images were collected with an Olympus Fluoview confocal laser scanning microscope (LSCM) FV300 (Olympus Corporation). For each image, the channels were collected sequentially with single wavelength excitation and then merged to produce the composite image using the software Fluoview v5.0. This avoids the problem of bleed-through of images resulting from side-band excitation of the fluorochromes. Brightness and contrast were adjusted in Adobe Photoshop 7.0. Images for Fig. 3 are displayed as negatives to permit visualization of faint fluorescent signal. This was accomplished by using the "Invert" command in Photoshop on the Red channel of the confocal image.

For quantification of immunocytochemical data, we counted immunoreactive cells from 3 sections through the vallate papilla from 2 different animals. An immunoreactive profile was included if it had an elongate morphology extending at least half the height of the taste bud and included an obvious nucleus. Cell fragments not including a nuclear profile were not included in the sample.

### RT-PCR analysis

Adult PLCβ2-GFP and GAD-GFP mice were killed by CO_2 _asphyxiation followed by decapitation, the tongue and palate were removed and a protease mixture consisting of 1 mg/ml collagenase, type A, 2.5 mg/ml dispase (both from Roche Products, Indianapolis, IN) and 1 mg/ml trypsin inhibitor (Sigma, St. Louis, MO) in Tyrode buffer was injected. Tyrode buffer consisted of, in mM: 139 NaCl, 5 KCl, 2 CaCl_2_, 1 MgCl_2_, 10 Hepes, 10 glucose, 10 Na pyruvate, and 5 NaHCO_3_; pH 7.2, 318–323 mOsm. Epithelium was peeled from underlying tissue after 20 min and incubated in Ca/Mg-free Tyrode's solution for 14 min. For Ca-Mg-free Tyrode buffer, CaCl_2 _and MgCl_2 _were replaced with 2 mM each EGTA and BAPTA.

To avoid contamination of samples with non-taste cells, taste buds from vallate, foliate, fungiform papillae and palate were collected in two stages. First, they were extracted under a stereomicroscope from the epithelium using glass pipettes (inner diameter at tip, 80 μm) and transferred into Tyrode buffer. Next, individual taste buds were identified by GFP fluorescence under under 200× magnification and transferred to lysis buffer (Absolutely Nanoprep kit, Stratagene). Total RNA was purified with DNAse I digestion and first strand cDNA synthesized with Superscript III (Invitrogen, Carlsbad, CA). PCR was performed in 20 μl using cDNA of one taste bud as template per reaction. Positive and negative controls were run in parallel from master mixes. We designed PCR primers using published cDNA sequences for each gene. Because Gαq, α11 and α14 are highly homologous, we placed primers in the most divergent regions. Primers are listed in Table 2. PCR was performed for 35 cycles. Most primer pairs spanned at least one intron to avoid amplifying genomic DNA. PCR products for all genes were validated by DNA sequencing.

**Table 2:** Primers for RT-PCR

**Protein & Gene**		**Accession #**	**Forward Primer (5'→3')**	**Reverse Primer (5'→3')**	**Product bp**	**Anneal °C**
Gα14	*Gna14*	NM_008137	*attagctacttcccagagtacaca*	*gctcagatcaccctctgtct*	256	62°C

			* tcatgcaacagagggacttg	* agggccatgctcaattacac	294	60°C

Gαq	*Gnaq*	NM_008139	*gtcgactacttcccagaatatgat*	*agtccaggacggcaataaat*	333	62°C

			* aacacacaccatccgtcaga	* ggcaagcagtggtctctagc	229	60°C

Gα11	*Gna11*	NM_010301	*agcccaagtcctgagtttga*	*tgccaagtcagagtggagaa*	236	60°C

Gαgus	*Gnat3*	NM_001081143	*gcaaccacctccattgttct*	*agaagagcccacagtctttgag*	286	58°C

PLCβ2	*Plcb2*	NM_177568	*gagcaaatcgccaagatgat*	*ccttgtctgtggtgaccttg*	163	60°C

SNAP25	*Snap25*	NM_011428	*ggcaataatcaggatggagtag*	*agatttaaccacttcccagca*	310	58°C

T1R2	*Tas1r2*	NM_031873	*aagcatcgcctcctactcc*	*ggctggcaactcttagaacac*	114	58°C

T1R3	*Tas1r3*	NM_031872	*gaagcatccagatgacttca*	*gggaacagaaggacactgag*	283	58°C

TrpM5	*Trpm5*	NM_020277	*gtctggaatcacaggccaac*	*gttgatgtgccccaaaaact*	234	58°C

β-actin	*Actb*	NM_007393	*ccctgtgctgctcacc*	*gcacgatttccctctcag*	328	58°C

Primers marked * (Gα14 and Gαq) are located closer to the mRNA 3' end and were only used on amplified RNA from pools of GFP-expressing cells (Fig. 2).

### Single-cell RT-PCR

Vallate taste buds were collected from PLCβ2-GFP mice (as above), then cells were dissociated by gentle trituration. GFP-labeled single cells were collected each into 60 μl of lysis buffer containing 200 ng of poly-Inosinic acid as a carrier, processed for RNA purification and cDNA synthesis. For preliminary analyses of cell type specific expression (Fig. 2), we collected individual GFP-labeled vallate taste cells, pooled them in lysis buffer and isolated RNA and linear amplified it as previously described [29]. The aRNA (amplified RNA) was subjected to reverse transcription and 0.1% of the cDNA was used as template for RT-PCR (40 cycles). For analyses of single cells, mRNA from each cell was reverse transcribed and then each 20 μl single cell cDNA was divided as follows: 5% each for β-actin and TrpM5, 10% each for Gαgus and Gα14, 20% each for T1R2 and T1R3 and 30% for Gαq. PCR was performed in 20 μl for 40 cycles.

### Quantitative RT-PCR

Quantitative RT-PCR was carried out as previously described[57] using the same primers as for end point PCR, and SYBR Green PCR mix (Bio-Rad) in a Bio-Rad iQ iCycler. Three independent samples of CV taste buds and of adjacent non-taste epithelium were purified and analyzed in parallel. The concentration of each mRNA was compared to a standard curve generated from a sequence-validated template and calculated using MyiQ software (Bio-Rad). All mRNA concentrations were normalized to β-actin mRNA which was run in parallel.

## Authors' contributions

MT carried out immunocytochemistry, performed histological analysis and documentation and characterized the Gα14 antiserum. GD performed the molecular studies including RT-PCR, quantitative RT-PCR and single cell RT-PCR-based profiling. MT and GD carried out the bulk of the experimental work for this project and contributed equally to the project. SK maintained and genotyped the Gαq-null mice, prepared tissue from Gαq-KO and paired WT animals for histological analysis. JB assisted with the immunocytochemical preparations and TEF assisted with documentation and analysis. NC and TEF supervised the molecular and histological studies respectively, and were instrumental in the conceptual design and analysis of the data. All authors contributed to writing and editing the manuscript.

## Supplementary Material

Additional file 1 **Gq/11/14 and Gagus staining in vallate taste buds in a TrpM5-GFP mouse.** Single confocal image planes from the image shown in Fig. 3A; TrpM5-GFP (**B**, green), Gagus (**D**, blue) and Gq/11/14 (**F**, red), respectively. Co-localization in single plane images of TrpM5-GFP and Gagus (**A**), TrpM5-GFP and Gq/11/14 (**C**), Gagus and Gq/11/14 (**E**) in the vallate papilla. Panel **G **shows the the z-stack of the combined Gαgus and Gq/11/14 images equivalent to text Fig. 3A. TrpM5 is a marker of Type II taste cells. Gq/11/14 stains about half (14 of 27) of the TrpM5-GFP cells. Usually the Gq/11/14 immunoreactive cells are different from those positive for Gagus. Scale bar 20 μM. Click here for file

Additional file 2 **Gaq/11/14 and PGP9.5 staining in TrpM5-GFP mouse.** Single confocal image planes from the image shown in Fig. 3B; TrpM5-GFP (B, green), Gq/11/14 (D, red) and PGP9.5 (F, blue), respectively. Co-localization in single plane images of TrpM5-GFP and Gq/11/14 (A), TrpM5-GFP and PGP9.5 (C), PGP9.5 and Gq/11/14 (E) in the vallate papilla. In G the z-stack of the entire E panel single plane images. PGP9.5, a marker of Type III cells, does not co-localize with either Gq/11/14 or TrpM5. Scale bar 20 μM. Click here for file

Additional file 3 **Gq/11/14 and Gagus staining in T1R3-GFP mouse**. Single confocal image planes from the image shown in Fig. 5; T1R3-GFP (B, green), Gq/11/14 (D, red) and Gagus (F, blue), respectively. Co-localization in single plane images of TrpM5-GFP and Gq/11/14 (A), TrpM5-GFP and Gagus (C), Gagus and Gq/11/14 (E) in the vallate papilla. The Gq/11/14 antibody stains most of T1R3-GFP cells (30 of 41). Fewer cells are strongly positive for Gagus (blue). The large majority of Gq/11/14-IR cells (30 of 35) exhibit T1R3-GFP. Only about half of Gagus IR cells show T1R3-GFP expression. Scale bar 20 μM. Click here for file
