# Supplementary figures and images for: Expression of Galpha14 in sweet-transducing taste cells of the posterior tongue
Source: BMC Neurosci. 2008 Nov 13;9:110. doi: 10.1186/1471-2202-9-110 (PMC2596171; doi:10.1186/1471-2202-9-110)

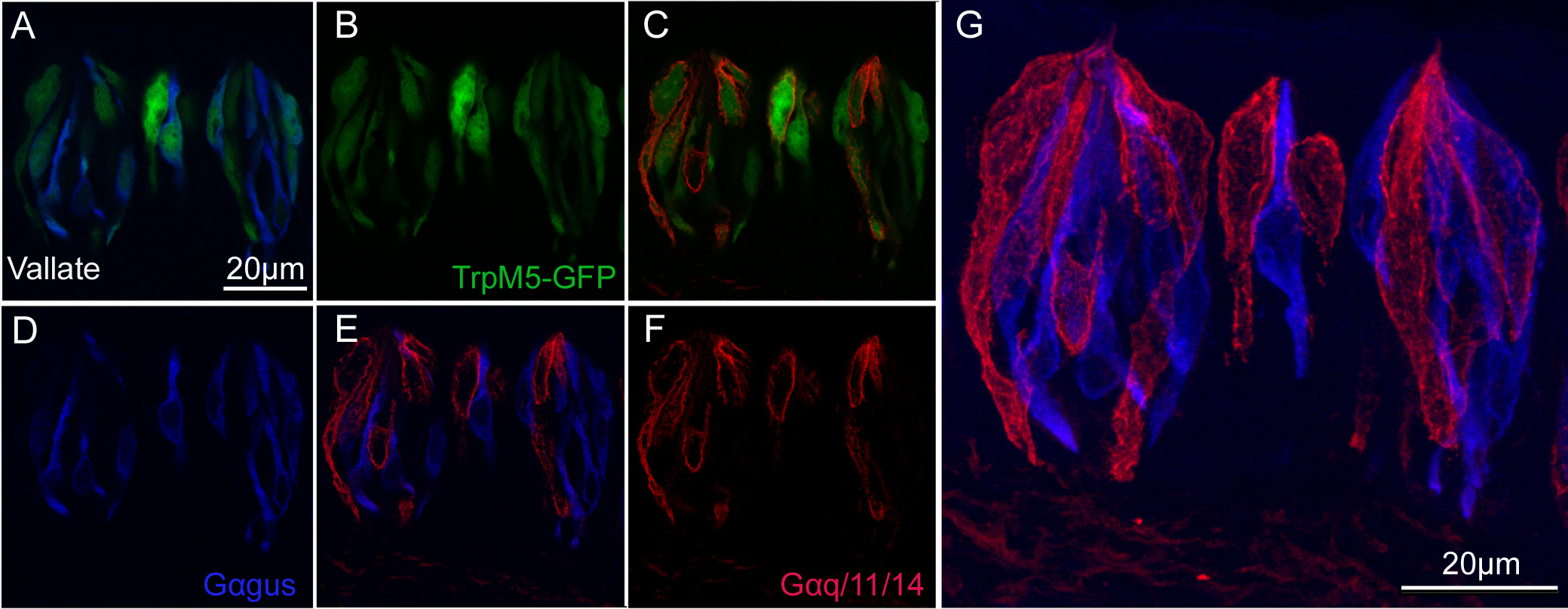

Supplement: Additional file 1 — Gq/11/14 and Gagus staining in vallate taste buds in a TrpM5-GFP mouse. Single confocal image planes from the image shown in Fig. 3A; TrpM5-GFP (B, green), Gagus (D, blue) and Gq/11/14 (F, red), respectively. Co-localization in single plane images of TrpM5-GFP and Gagus (A), TrpM5-GFP and Gq/11/14 (C), Gagus and Gq/11/14 (E) in the vallate papilla. Panel G shows the the z-stack of the combined Gαgus and Gq/11/14 images equivalent to text Fig. 3A. TrpM5 is a marker of Type II taste cells. Gq/11/14 stains about half (14 of 27) of the TrpM5-GFP cells. Usually the Gq/11/14 immunoreactive cells are different from those positive for Gagus. Scale bar 20 μM. [file 1471-2202-9-110-S1.jpeg]

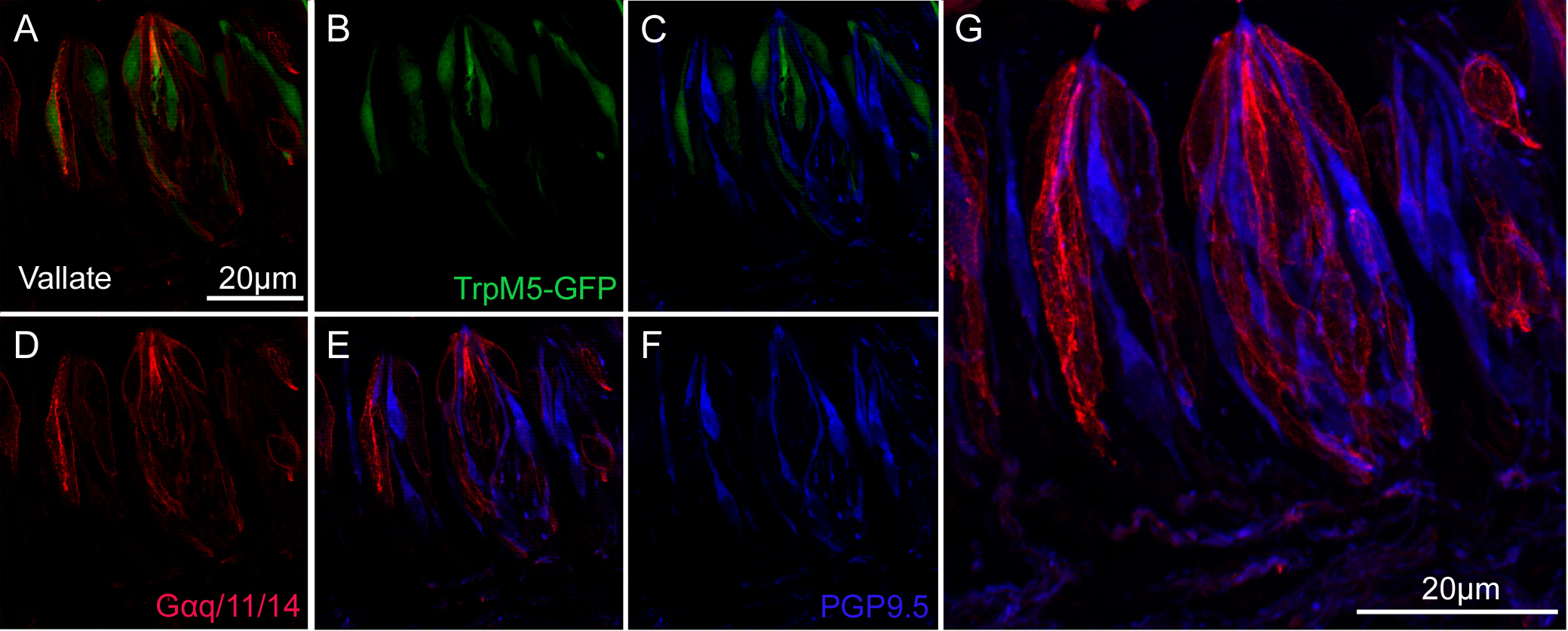

Supplement: Additional file 2 — Gaq/11/14 and PGP9.5 staining in TrpM5-GFP mouse. Single confocal image planes from the image shown in Fig. 3B; TrpM5-GFP (B, green), Gq/11/14 (D, red) and PGP9.5 (F, blue), respectively. Co-localization in single plane images of TrpM5-GFP and Gq/11/14 (A), TrpM5-GFP and PGP9.5 (C), PGP9.5 and Gq/11/14 (E) in the vallate papilla. In G the z-stack of the entire E panel single plane images. PGP9.5, a marker of Type III cells, does not co-localize with either Gq/11/14 or TrpM5. Scale bar 20 μM. [file 1471-2202-9-110-S2.jpeg]

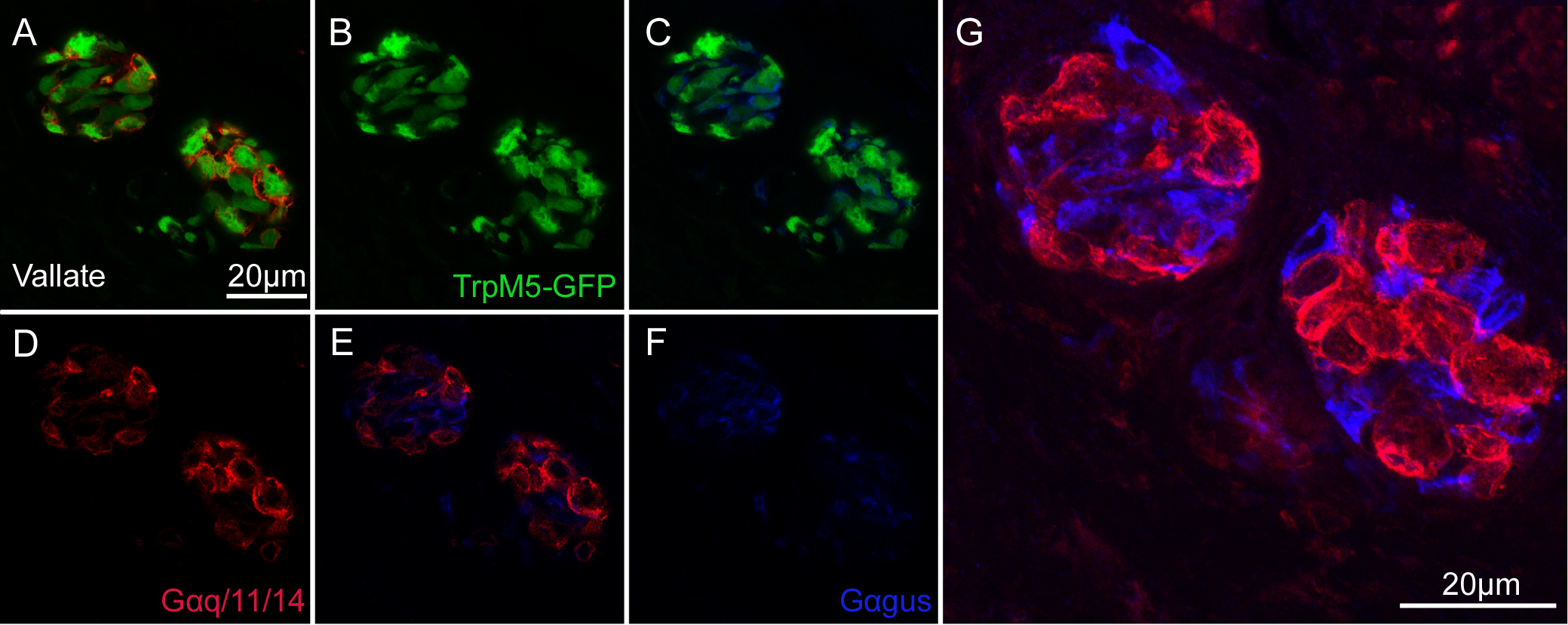

Supplement: Additional file 3 — Gq/11/14 and Gagus staining in T1R3-GFP mouse. Single confocal image planes from the image shown in Fig. 5; T1R3-GFP (B, green), Gq/11/14 (D, red) and Gagus (F, blue), respectively. Co-localization in single plane images of TrpM5-GFP and Gq/11/14 (A), TrpM5-GFP and Gagus (C), Gagus and Gq/11/14 (E) in the vallate papilla. The Gq/11/14 antibody stains most of T1R3-GFP cells (30 of 41). Fewer cells are strongly positive for Gagus (blue). The large majority of Gq/11/14-IR cells (30 of 35) exhibit T1R3-GFP. Only about half of Gagus IR cells show T1R3-GFP expression. Scale bar 20 μM. [file 1471-2202-9-110-S3.jpeg]
